# Targeted *in vitro* gene silencing of E2 and nsP1 genes of chikungunya virus by biocompatible zeolitic imidazolate framework

**DOI:** 10.3389/fbioe.2022.1003448

**Published:** 2022-12-14

**Authors:** Rajarshee Tagore, Kalichamy Alagarasu, Poonam Patil, Suneela Pyreddy, Shakil Ahmed Polash, Mahadeo Kakade, Ravi Shukla, Deepti Parashar

**Affiliations:** ^1^ Dengue and Chikungunya Group, ICMR-National Institute of Virology, Pune, India; ^2^ Ian Potter NanoBioSensing Facility, NanoBiotechnology Research Laboratory, School of Science, RMIT University, Melbourne, VIC, Australia; ^3^ Centre for Advanced Materials and Industrial Chemistry, RMIT University, Melbourne, VIC, Australia

**Keywords:** chikungunya virus, gene silencing, metal-organic framework, zeolitic imidazolate frameworks, siRNA

## Abstract

Chikungunya fever caused by the mosquito-transmitted chikungunya virus (CHIKV) is a major public health concern in tropical, sub-tropical and temperate climatic regions. The lack of any licensed vaccine or antiviral agents against CHIKV warrants the development of effective antiviral therapies. Small interfering RNA (siRNA) mediated gene silencing of CHIKV structural and non-structural genes serves as a potential antiviral strategy. The therapeutic efficiency of siRNA can be improved by using an efficient delivery system. Metal-organic framework biocomposits have demonstrated an exceptional capability in protecting and efficiently delivering nucleic acids into cells. In the present study, carbonated ZIF called ZIF-C has been utilized to deliver siRNAs targeted against E2 and nsP1 genes of CHIKV to achieve a reduction in viral replication and infectivity. Cellular transfection studies of E2 and nsP1 genes targeting free siRNAs and ZIF-C encapsulated siRNAs in CHIKV infected Vero CCL-81 cells were performed. Our results reveal a significant reduction of infectious virus titre, viral RNA levels and percent of infected cells in cultures transfected with ZIF-C encapsulated siRNA compared to cells transfected with free siRNA. The results suggest that delivery of siRNA through ZIF-C enhances the antiviral activity of CHIKV E2 and nsP1 genes directed siRNAs.

## Introduction

Chikungunya Virus (CHIKV), the causative agent of chikungunya fever belongs to the genus *Alphavirus* of family *Togaviridae* (Pialoux et al., 2007). CHIKV is transmitted through the bite of an infected *Aedes albopictus* or *Aedes aegypti* mosquito. Symptoms of this arboviral disease include fever, headache, arthralgia, myalgia, and a maculopapular rash (or sometimes a petechial rash). After the bite of an infected mosquito, the onset of illness usually occurs 3–7 days later (but can range from 2–12 days) (Parashar et al., 2013). Studies revealed the recurrence of polyarthritis several years after the initial infection emphasizing the degree of severity of chikungunya fever as a public health concern in tropical countries in Africa, Asia, central and south America (Suhrbier, 2019). The recent re-emergence of CHIKV on an epidemic scale in temperate countries of North America and Europe shows its importance as a potent health hazard (Schwartz and Albert, 2010; Rougeron et al., 2015). Currently, there is no effective vaccine or therapy to target the virus, and treatment plans mainly involve supportive treatment using analgesics along with the administration of non-steroidal anti-inflammatory drugs (NSAIDs).

RNA interference (RNAi) has come up as a potential strategy for antiviral therapy against multiple viruses (Sharabati et al., 2022). It involves the post-transcriptional silencing of the target genes by the RNA-induced silencing complex *via* corresponding mRNA degradation in the cytosol. Small interfering RNAs (siRNAs) are small oligonucleotides of length 18–23 bp which are synthesized with a sequence homologous to that of the target gene(s) and serve as one of the two main facets of RNAi (Tan and Yin, 2004; Zhuang et al., 2020). Recent studies have indicated that the introduction of siRNA directly into cells serves as a potential antiviral therapy by inhibiting viral replication and gene expression for RNA viruses like CHIKV, Influenza, Human Immunodeficiency Virus (HIV), hepatitis, Japanese Encephalitis, etc (Ge et al., 2003; Kapadia et al., 2003; Lee and Rossi, 2004; Murakami et al., 2005; Parashar et al., 2013). Despite showing credible success, there are several hurdles that impede the use of RNAi as a therapeutic strategy on a larger scale. Some of those problems include lower cellular uptake and knockdown efficiency due to rapid degradation of administered RNAi by nucleases. In addition, possible immunogenicity, rapid filtration, and clearance from blood circulation also serve as limiting factors for therapeutic efficacy (Castanotto and Rossi, 2009). Therefore, the use of nanoparticles for delivering siRNA can overcome the aforementioned hurdles and may serve as a potential strategy to increase the bioavailability of the siRNA (Yin et al., 2014). Nanoparticles could prevent the degradation of the siRNA from endogenous ribonucleases and their surface could be modified by external coatings with membranes or other ligands to achieve tissue specific delivery (Liang et al., 2015; Lorenzer et al., 2015).

Metal-organic frameworks (MOFs) are a class of porous and crystalline materials that are formed *via* a coordination bonds between metal ions which act as a node and organic ligands which act as bridges to connect these nodes. Unique chemical composition, alterable pore sizes, and high surface area of MOFs enables its use for catalysis (Liu et al., 2014; Huang et al., 2017), gas storage and separation (Li et al., 2009), chemical separation (Khan and Jhung, 2017), etc. MOFs have great potential in biomedical applications due to their exceptional properties such as high porosity, large pore size, extensive surface area, biocompatibility, and biodegradability (Sharabati et al.,2022). Scaling down the size of these MOF particles to the nanoscale has unraveled their use as a potent and effective delivery system providing a wide range of applications like drug delivery, imaging, photothermal therapy, etc. (Huxford et al., 2010; Giménez-Marqués et al., 2016; Lakshmi and Kim, 2019; Luo et al., 2019). Till now, a wide range of drugs, cellular proteins, and enzymes have been reportedly delivered successfully utilizing different varieties of MOFs (Velásquez-Hernández et al., 2021). A few examples of such drugs and proteins are doxorubicin (Zhang et al., 2017), cisplatin (Taylor-Pashow et al., 2009), ceftazidime (Sava Gallis et al., 2019), naringin (Yu et al., 2017), insulin (Yang et al., 2020), gemcitabine monophosphate (Li et al., 2020), tyrosinase (Lian et al., 2018), etc. Delivery of oligonucleotides as a part of nucleic acid-based therapy has also been reported (Morris et al., 2014; Chen et al., 2017; Li et al., 2019; Poddar et al., 2019; Su et al., 2019; Poddar et al., 2020). Zeolitic imidazolate frameworks (ZIF) are a subcategory of MOFs made of metal ions (Zn, Cu, Co., Fe, etc.) connected by imidazolate linkers which have been used for multiple purposes, the primary one being a drug carrier (Chen et al., 2014). ZIF-8 is a rigorously studied and frequently used member of the ZIF family of MOFs. It is composed of zinc ions and 2-methylimidazolate. A study reported the antiviral activity of ZIF-8 on CHIKV (Chandra et al., 2019). ZIF-8 demonstrates high biocompatibility and low cytotoxicity combined with high thermal and hydrothermal stability and has been used as a suitable nanocarrier for various drugs (Park et al., 2006; Hoop et al., 2018). ZIF-8, though stable under physiological conditions, is unstable in acidic environments and undergoes degradation; a property that has been exploited successfully for drug delivery. ZIF-8 has already been reported to be a successful nanocarrier for gene therapeutic agents like siRNA, plasmids, oligonucleotides etc. (Poddar et al., 2019; Abdelhamid et al., 2020a; Zhuang et al., 2020). The transfection efficiency of such gene therapeutic agents increases manifold when conjugated with inorganic nanomaterials which allows its usage in several combinations for a versatile treatment plan (Abdelhamid et al., 2020b). The instability of well-studied ZIF-8 in water motivated us to find water-favoured ZIF for cell-based studies (Zhang et al., 2015; Zhang et al., 2019). However, the usage of ZIFs as a carrier for antiviral purposes has not been investigated extensively as of yet.

Though, CHIKV genome possesses several targets as means of antiviral therapy, the non structural protein 1 (nsP1) and envelope 2 (E2) proteins are more attractive targets since nsP1 is responsible for RNA synthesis and capping whereas the structural E2 glycoprotein mediates interaction with the host cell surface receptors (Parashar et al., 2013). Their role in infection and multiplication inside host cells makes them a prime target for antiviral therapy (Wong and Chu, 2018). Hence, the primary objective of this study is to analyze the efficiency of ZIF-C as a potential delivery system for the delivery of siRNA targeted against E2 and nsP1 genes of the CHIKV and to study the extent of RNAi observed.

## Materials and methods

### Cells, virus, and chemicals

Vero CCL-81 cell line (ATCC^®^ CCL 81 derived from the kidney of African green monkey) was chosen as the host and CHIKV strain of African genotype (Strain No. 061573; Andhra Pradesh 2015) was used (Yergolkar et al., 2006). Modified Eagle’s Medium (MEM) supplemented with 10% Foetal Bovine Serum (FBS) (Gibco, Technologies, NY, United States) and anti mycotic antibiotic solution (Sigma Aldrich, United States) was used to maintain the Vero CCL-81 cell line. Throughout the study, 0.01 Multiplicity of Infection (MOI) of virus stock was used for infection. 2-methyl imidazole (Sigma-Aldrich, United States) and zinc acetate dihydrate (Sigma-Aldrich, United States) were used for the synthesis of ZIF-C bio-composites.

### siRNA

siRNA targeted against E2 and nsP1 genes (designated as Chik1 and Chik5, respectively) were designed using HP OnGuard siRNA design and synthesized (Qiagen, Germany) as described previously (Parashar et al., 2013). The minimum effective dose of siRNA as was investigated earlier to be 400 pM (Jeengar et al., 2022), was used during the synthesis of siRNA encapsulated ZIF-C nanoparticles.

### Synthesis of siRNA encapsulated ZIF-C bio-composites

The nano bio-composites were prepared following protocols published earlier (Poddar et al., 2020). Briefly, fresh solutions of 2-methyl imidazole (2 mIM, 160 mM) and zinc acetate dihydrate (40 mM) were prepared in ultrapure/miliQ water. Chik1 siRNA and Chik5 siRNA at 400 pM concentration each and a combination of both siRNAs containing 400 pM of each type were added in separate tubes to mix with 100 µL of 2 mIM. Next, zinc acetate dihydrate was added (100 µL) to each mixture. The tubes were incubated at room temperature for 10 min under static conditions. The tubes were centrifuged at 10,000 g for 10 min and the formed pellets were washed with water thrice to remove unreacted precursors. The produced bio-composites were named Chik1-ZIF-C, Chik5-ZIF-C, and Chik1,5-ZIF-C, respectively, for further reference.

### Characterization of siRNA-ZIF-C bio-composites

X-ray diffraction (XRD) was performed in Bruker D8 General Area Detector Diffraction System (GADDS) using Cu Kα (λ = 1.54056 Å) radiation at 40 kV generator intensity and 40 mA current to determine the crystalline property of Chik1-ZIF-C. The 2*θ* scanning range was 5–40° and the step size was 0.01°C. The vacuum-dried samples were mounted on a clean silicon wafer attached to the sample holder and placed into the instrument. The spectra were recorded at room temperature. The acquired raw data (raw) were converted to a UXD file using File Exchange Program XCH (Ver. 5.0.10, 2004, Bruker AXS, Socabim, Karlsruhe, Germany). Following that, the file was converted to Text Documents (.txt) and plotted using the OriginPro software. FEI Verios 460 L Scanning Electron Microscope (SEM) was used to characterize the morphology of Chik1-ZIF-C. The accelerating voltage and current of the electron beam were 3 kV and 0.2 nA, respectively. The diluted biocomposite was drop-casted on a clean silicon wafer surface and air-dried to remove the solvent. A 5 mm iridium coating was applied using Leica EM ACE600 Sputter Coater to enhance conductivity and vacuum durability in the SEM chamber. The Transmission electron microscopy (TEM) images of Chik1-ZIF-C is a taken in JEOL 1010 TEM operated at 100 kV. Fourier transform infrared (FT-IR) spectroscopy was carried out to reveal the functional groups in Chik1-ZIF-C. Potassium bromide (KBr) was added to the vacuum-dried pellet and the mixture was kept at 60°C for 1 h to remove all moisture. Then, the thoroughly mixed KBr-pellet mixture was transferred to the sample holder. Pure KBr was run initially as a background. The spectrum was obtained in the range 4,000–400 cm^−1^ using PerkinElmer Frontier^™^. An average of 128 scans was recorded at a solution of 4 cm^−1^. Raman spectrum was recorded in PerkinElmer Raman Station 400 using a laser diode, of which the excitation wavelength was 785 nm under atmospheric conditions. Before the sample run, the sample was dried overnight at room temperature to remove any residual water before the analyses. Then the sample was placed on a clean glass slide and placed on the sample holder of the instrument. The laser irradiated sample was acquired with a 1 cm^−1^ spectral resolution. OPTIZEN NanoQ was used to determine the siRNA loading efficiency of ZIF-C. The bioanalytical system was calibrated with molecular-grade purified water and recorded the amount of siRNA available in the collected supernatant. This value was subtracted from the initial siRNA value to get the loading efficiency of ZIF-C. Finally, the concentration of the prepared biocomposites was determined from Microwave Plasma Atomic Emission Spectroscopy (MP-AES) using standard solutions of Zn (0–40 ppm) prepared in 2% nitric acid (HNO_3_). MOF bio-composites were digested in concentrated HNO_3_ for about an hour and the final volume was adjusted with milliQ water to keep the same acid strength (i.e., 2% HNO_3_). The concentration of ZIF-C was measured based on Zn-equivalent.

### Evaluation of cytotoxicity of siRNA ZIF-C bio-composites nanoparticles on vero CCL-81 cells

The effect of ZIF-C nanoparticles on Vero CCL-81 at different time points was studied using the 3-(4,5-dimethylthiazol-2-yl)-2,5-diphenyl-2H-tetrazolium bromide (MTT) assay. Vero CCL-81 cells were seeded in a 96-well microtitre plate at a density of 35,000 cells per well. Cells were then treated with Chik1-ZIF-C and Chik5-ZIF-C, each formulation with concentration of siRNA ranging from 100–1,000 pM, in serum-free MEM for 6 h and was replaced by MEM with 2% FBS and was incubated at 37°C with 5% CO_2_ till completion of 24 h. After 24 h, 10 µL of MTT solution (5 mg/ml) was added to each well and incubated at 37°C for 3 h. The cells were then treated with 100 µL of acidified isopropanol (5% 0.1 N HCl in isopropanol) and incubated for 1 h at 37°C. The readings were taken in a microplate reader at a wavelength of 570 nm with a reference filter at 690 nm. Percent viability was calculated in comparison with cells untreated with nanoparticles. Cytotoxicity assays were also perfomed for lipofectamine 2000 formulations with siRNA concentrations ranging from 100–1,000 pM. The concentration of lipofectamine 2000 used was 12 ng/μl as indicated by manufacturer.

### Assessment of RNAi in CHIKV infected ZIF-C treated cells

Chik1-ZIF-C, Chik5-ZIF-C, and Chik1,5-ZIF-C were assessed for antiviral effects on cells after infection. Vero CCL-81 cells were seeded in 24 well plates at a density of 2 × 10^5^ cells per well and incubated at 37°C at 5% CO_2_ for 24 h. The cells were then infected with 0.01 MOI CHIKV for 1 h. Later, cells were treated with Chik1-ZIF-C and Chik5-ZIF-C, with siRNA concentrations of 50, 100, 200, 400 and 800 pM immediately after infection or 0 h post-treatment (h.p.i) for 6 hours after which MEM with siRNA formulations was replaced by MEM with 2% FBS. To find out whether the siRNA effect is sequence specific, experiments with siRNA against SARS-CoV-2 siRNA was also perfomed (Panda et al., 2021a). For further experiments, 400 pM of Chik-ZIF-C siRNA formulation was chosen. Post infection, cells were treated with 400 pM of Chik1-ZIF-C, Chik5-ZIF-C, and Chik1,5-ZIF-C formulations for six hours at three different time points i.e., 0 h post-infection (h.p.i), 3 h. p.i, and 6 h. p.i. After treatment, MEM with siRNA formulations was replaced by MEM with 2% FBS and incubated until the completion of 24 h. Lipofectamine mediated transfection of the same combinations of siRNA (400 pM) was also performed as a control. The combinations were simply named Chik1-Lipo, Chik5-Lipo, and Chik1,5-Lipo for ease of reference. Virus control (VC) comprised of wells containing untreated virus infected cells while cell control (CC) comprised uninfected untreated cells. An additional group of control wells was maintained in which infected cells were treated with ZIF-C alone. In all conditions, plates were frozen at -80 °C and thawed to collect cell supernatant for quantitative estimation of viral RNA by real-time quantitative reverse transcription PCR (qRT-PCR) and estimation of infectious virus titre by focus forming unit assay (FFU). The effect of ZIF-C mediated siRNA delivery on percent of virus infected cells was assessed by immunofluorescence assay (IFA). All the experiments were performed in triplicates.

### Focus forming unit assay

FFU assay was performed as reported earlier (Panda et al., 2021b; Patil et al., 2021) with modifications. The primary antibody used was an in-house developed mouse anti-chikungunya antibody (1:300 dilution), while the secondary antibody was a goat anti-mouse IgG Horseradish peroxidase (HRP) conjugate (1:1,000 dilution) [Sigma-Aldrich St. Louis, MO, United States].

#### Immunofluorescence assay

In a 24-well plate (Tissue Culture Test Plate 24, TPP, Switzerland), Vero CCL-81 cells were seeded at a density of 2 × 10^5^ cells per well with a coverslip placed in each well. After the formation of a confluent monolayer, the cells were infected with CHIKV and treated with different formulations of siRNA i.e., Chik1-ZIF-C, Chik5-ZIF-C, Chik1,5-ZIF-C, Chik1-Lipo, Chik5-Lipo, and Chik1,5-Lipo and incubated for 12 h. Following incubation, the IFA was carried out as described in a previous study (Parashar et al., 2013). The in-house developed mouse anti-chikungunya antibody was used as a primary antibody. The secondary antibody used was conjugated with FITC fluorophore. DAPI was used to stain the nucleus of the cells.

To determine the proportion of infected cells, total cells and infected cells were counted in four randomly selected fields per coverslip, and the percent of infection was calculated by dividing the number of infected cells in a field by the total number of cells in that field and multiplying it with 100.

### Quantitative reverse transcription polymerase chain reaction (qRT-PCR)

The viral RNA copy number was quantified using a qRT-PCR assay. Using a commercial RNA extraction kit (Qiagen, Hilden, Germany), viral RNA was extracted from Vero CCL-81 cell culture supernatants. qRT-PCR was performed in a single step as previously reported with primers targeting the E3 gene (Parashar et al., 2013). The sample’s viral RNA copy number was calculated using a standard curve generated with *in-vitro* transcribed viral RNA standards.

## Results

### Biocomposite characterization

**FIGURE 1:**
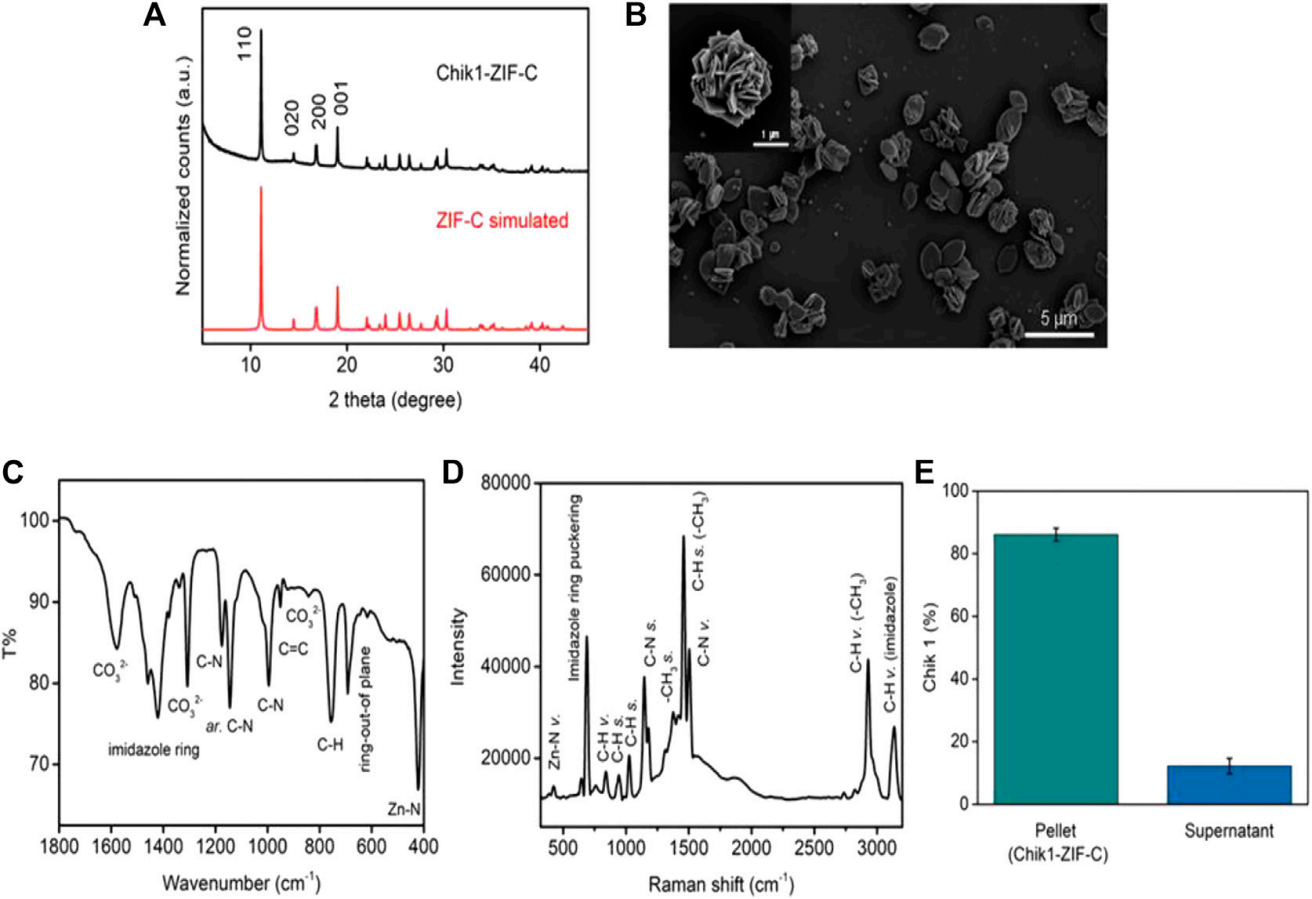
Characterization of Chik1-ZIF-C. **(A)** XRD of Chik1-ZIF-C was compared with simulated ZIF-C; **(B)** morphology was revealed by SEM; functional chemical groups and surface composition were studied by **(C)** FT-IR and **(D)** Raman spectroscopy; **(E)** quantification of encapsulated Chik1 sequence within ZIF-C MOF pellet.

The solution of nucleotide and imidazole mixture turned cloudy soon after the addition of zinc salt. The formed biocomposite was collected by centrifugation and the pellet was washed thrice with water to remove any unreacted ions. The X-Ray Diffraction (XRD) pattern of Chik1-ZIF-C (Figure 1A) revealed the high crystallinity of the structure. A simulated ZIF-C pattern was used from an earlier study to identify the correct ZIF phase for comparison (Basnayake et al., 2015). The diffracted peaks at 11.03, 14.35, 16.95, 18.31, 22.12, and 23.33^o^ positions matched perfectly with the simulated value. No additional peaks were observed, and it confirmed that the ZIF-C biocomposite preparation was pure. The typical plate-like morphology of siRNA-loaded ZIF-C was observed under SEM (Figure 1B and Supplementary Figure S1) similar to previous studies reported for ZIF-C bio-composites (Carraro et al., 2020; Poddar et al., 2020). FT-IR spectroscopy (Figure 1C) provided further evidence on the formation of ZIF-C biocomposite. The band at 424 cm^−1^ is due to the Zn-N stretching of the framework (Hu et al., 2011). The band at 692 cm^−1^ is attributed to the ring of out-of-plane bending vibrations of the imidazole. The peaks at 996 and 760 cm^−1^ could be assigned to C-N and C-H bending vibrations, respectively. The intense peak at 1,148 cm^−1^ is derived from aromatic C-N stretching vibrations. Moreover, signals between 1,450-1,300 refer to entire ring stretching (Prabhu et al., 2011). Two strong bands (at 1,579 and 1,379 cm^−1^) and one medium band (at 842 cm^−1^) are attributed to the asymmetric stretching and bending mode of carbonate, respectively (Basnayake et al., 2015). Raman spectrum of Chik1-ZIF-C was plotted in Figure 1D **.** Bands at 288 and 688 cm^−1^ refer to Zn-N vibration and imidazole ring puckering, respectively. The 838 cm^−1^ band corresponds to C-H vibration. Peaks at 954 and 1,028 cm^−1^ are attributed to C-H stretching. In addition, bands at 1,148 and 1,182 suggest the presence of C-N stretching. Moreover, the 1,384 cm^−1^ band is due to methyl stretching and the 1,458 cm^−1^ band refers to the C-H stretch of the methyl group of imidazolate. The peak at 1,508 cm^−1^ is referred to as C-N vibration. Two peaks at 2,928 and 3,132 cm^−1^ suggested the presence of C-H vibration of the methyl group and C-H vibration of the imidazole ring. Taken together, these results confirm the typical characteristics and morphology of ZIF-C in the siRNA bio-composites. Moreover, the biocomposite pellet encapsulated maximum siRNA within 10 min of reaction, and about ∼87% of siRNA was loaded inside the framework (Figure 1E). Microwave Plasma Atomic Emission Spectroscopy (MP-AES) was used to determine the concentrations of Zn in the bio-composites which were found at ∼28 μg/ml (Supplementary Figure S2). Energy Dispersive X-ray (EDX) analysis was used to identify the elemental composition and existence of the elements C, N, Zn and O was found in Chik1-ZIF-C (Supplementary Figure S3).

### Effect of ZIF-C treatment on proliferation of vero CCL-81 cells (MTT assay)

**FIGURE 2:**
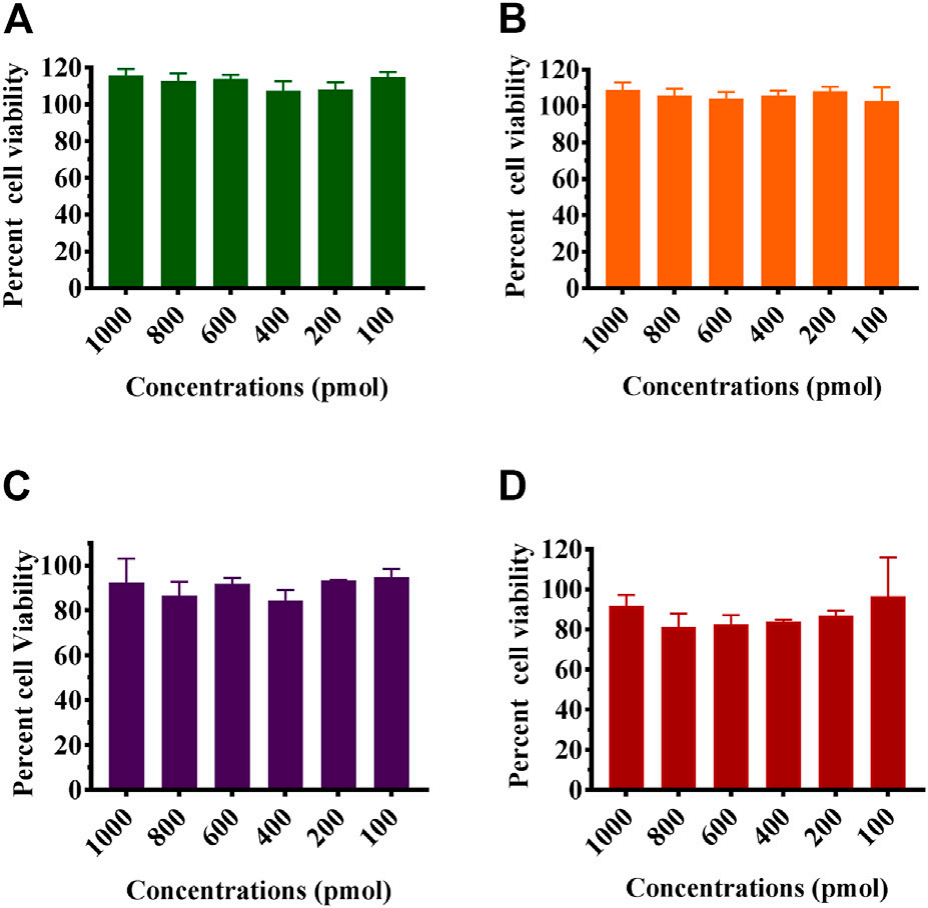
Effect of siRNA-ZIF-C (A and B) and lipofectamine (C and D) on the viability of cells using MTT assay. Vero CCL- 81 cells were treated with the ZIF-C siRNA formulations and lipofectamine siRNA formulations in serum free MEM i.e. Chik1 **(A–C)**, Chik5 **(B–D)** for 6 h and was replaced by MEM with 2% FBS. After incubation for 24 h post infection, MTT solution (5 mg/ml) was added to each well and incubated at 37°C for 3 h. The cells were then treated with acidified isopropanol and incubated for 1 h at 37°C followings which optical density was measured using a microplate reader and percent cell viability was calculated.

MTT assay was used to study the effect of the formulated Chik1-ZIF-C, Chik5-ZIF-C, Chik1-Lipo, and Chik5 on the viability of Vero CCL-81 cells (Figure 2). The results revealed that Chik1-ZIF-C and Chik5-ZIF-C formulations with different concentrations of siRNA were non-toxic (cell viability greater than 95%) (i.e. 9-10 μg/ml Zn equivalent) (Figures 2A, B). For lipofectamine siRNA formulations, the cell viability compared to ZiF-C siRNA formulations was less (∼80%) (Figures 2C, D).

### Effect of ZIF-C mediated siRNA delivery on CHIKV replication

**FIGURE 3:**
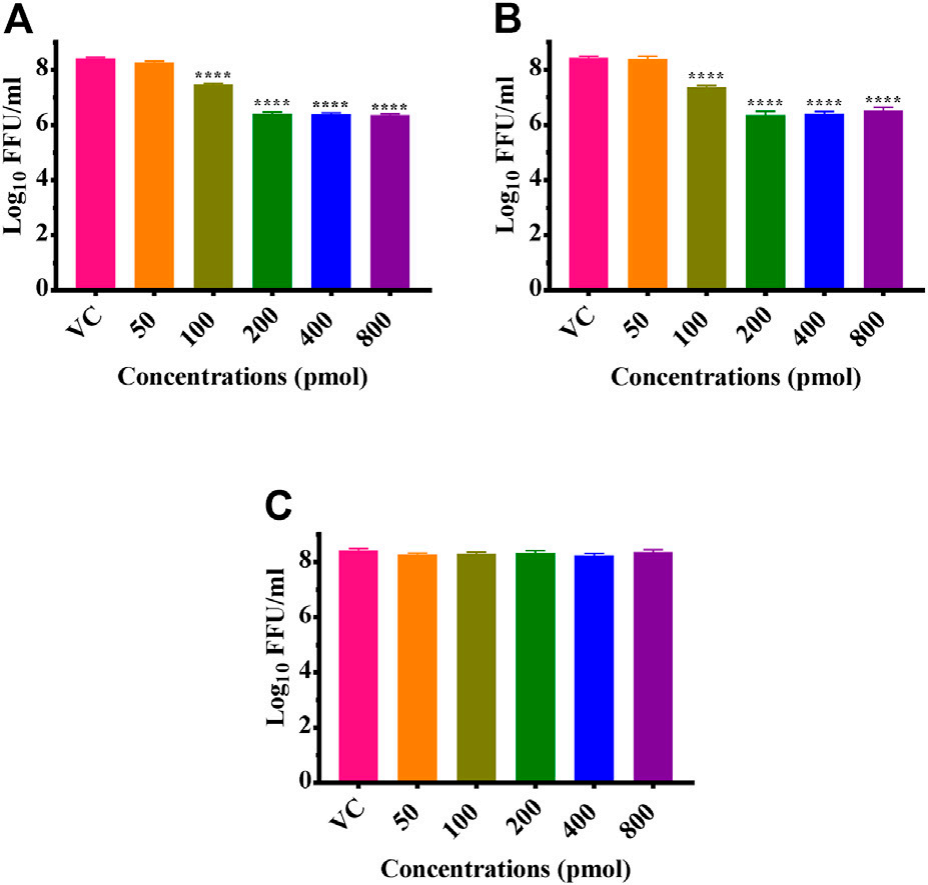
Dose dependent effects of ZIF-C siRNA formulations on CHIKV replication. Vero CCL- 81 cells were then infected with 0.01 MOI CHIKV for 1 h. Later, cells were treated with Chik1-ZIF-C **(A)**, Chik5-ZIF-C **(B)** and SARS-CoV-2-ZIF-C **(C)** immediately after infection for 6 hours and replaced by MEM with 2%FBS. The cells were incubated for 24 h post infection. Then plates were frozen and later FFU assay was done using the cell culture supernatant. Mouse anti-chikungunya antibody and goat anti-mouse IgG HRP conjugates were used as primary and secondary antibodies, respectively in FFU assay. The experiments were performed in triplicates. All the values are expressed as mean ± SEM. The FFU titres were compared between VC and different siRNA formulations using one-way ANOVA with multiple corrections (****p < 0.0001).

**FIGURE 4:**
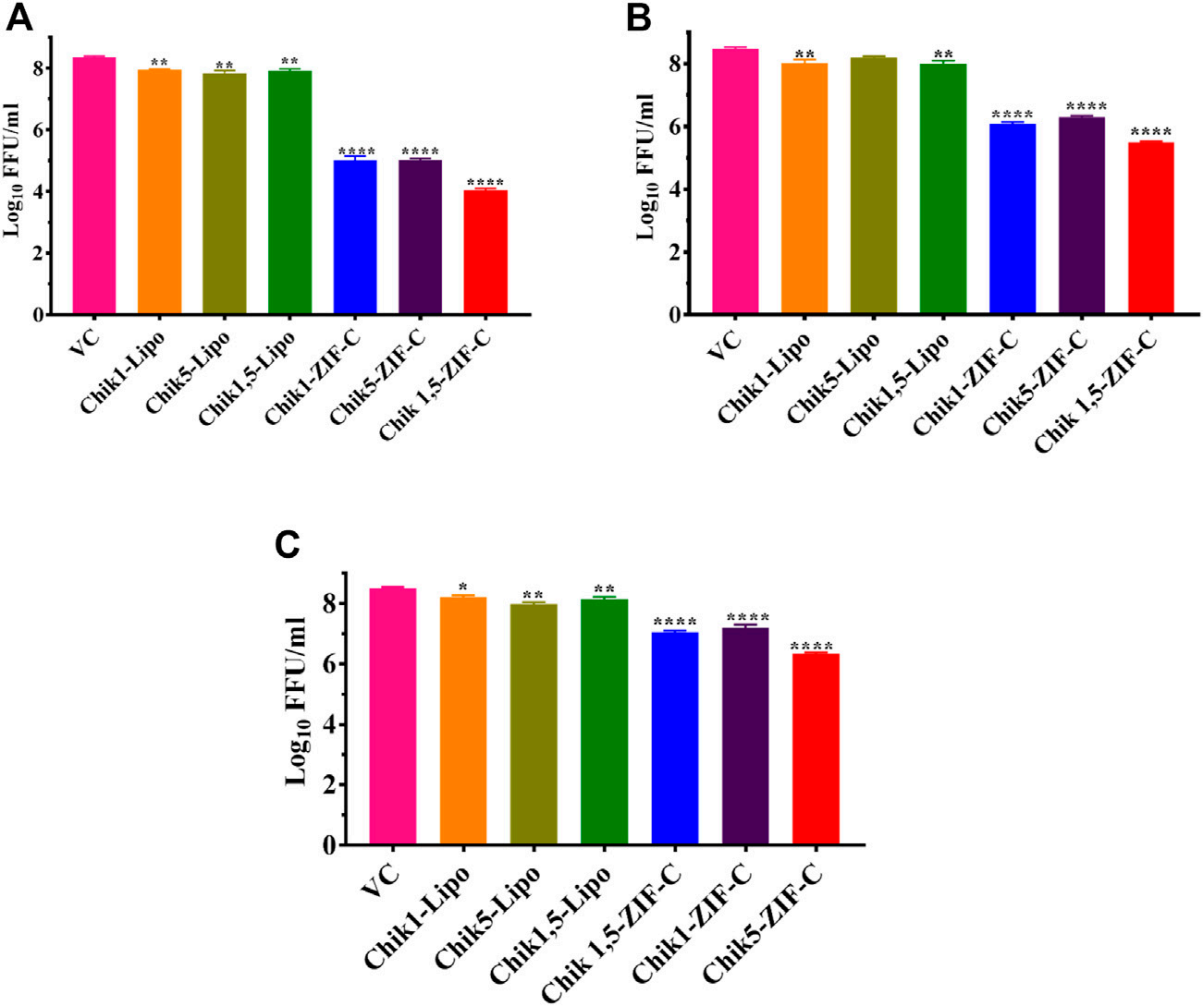
Time of addition dependent inhibitory effects of siRNA-ZIF-C and lipofectamine mediated delivery of siRNA on the production of virus particles. Vero CCL-81 cells were treated with the ZIF-C MOF siRNA formulations (Chik1-ZIF-C, Chik5-ZIF-C and Chik1,5-ZIF-C) and lipofectamine 2000 siRNA complexes (Chik1-Lipo, Chik5-Lipo, and Chik1,5-Lipo) at 0 **(A)**, 3 **(B)** and 6 **(C)** h.p.i. Irrespective of time of addition, the treatment was provided for 6 hours followed by replacement *w*th fresh MEM containing 2% FBS. The cells were incubated for 24 h post infection. Then the plates were frozen and later thawed to collect the cell culture supernatant to perform the FFU assay. The experiments were performed in triplicates in three independent trials. All the values are expressed as mean ± SEM. The FFU titres were compared between VC and different siRNA formulations using one-way ANOVA with multiple corrections (****p < 0.0001, ***p < 0.001, **p < 0.01, *p < 0.05).

CHIKV infected Vero CCL-81 cells were treated with either any of the ZIF-C formulations namely Chik1-ZIF-C, Chik5-ZIF-C, and Chik1,5-ZIF-C, or any of the lipofectamine formulations namely Chik1-Lipo, Chik5-Lipo and Chik1,5-Lipo at three different time-points post-infection (h.p.i.), i.e. 0, 3 and 6 h. p.i. to find out the efficacy of inhibition of replication of CHIKV by siRNA as delivered by ZIF-C and Lipofectamine 2000. The culture filtrate was assessed by FFU to find out the effect of different treatments on the infectious virus titre. A siRNA dose dependent reduction in virus titre was observed and the reduction was more or less similar in formulations with siRNA concentration ≥200 pM (Figures 3A,B). The IC50 value for both Chik1 and Chik5 was observed to be ∼0.029 pM ZIF-C-SARS-CoV-2 siRNA formulation had no effect on CHIKV titre (Figure 3C). Further experiments were performed with 400 pM concentration of siRNA and experiments were repeated with different time points of addition of ZIF-C siRNA formulations. A significant reduction in infectious virus titre was noticed across all three time points in the case of treatment with Chik1-ZIF-C, Chik5-ZIF-C, and Chik1,5-ZIF-C. There was a significant reduction in virus titre from 8.4 log_10_ FFU/mL in virus control (VC) to 5.0 log_10_ FFU/mL in Chik1-ZIF-C treated cells, 5.0 log_10_ FFU/mL in Chik5-ZIF-C treated cells and 4.1 log_10_ FFU/mL in Chik1,5-ZIF-C treated cells when the treatment was given at 0 h. p.i (*p* < 0.0001) (Figure 4A). Treatment of CHIKV infected Vero CCL-81 cells with Chik1-ZIF-C, Chik5-ZIF-C and Chik1,5-ZIF-C at 3 h. p.i (Figure 4B) resulted in a lower reduction of virus titre (as compared to treatment at 0 h. p.i) from 8.5 log_10_ FFU/mL in VC to 6.1 log_10_ FFU/mL, 6.3 log_10_ FFU/mL and 5.5 log_10_ FFU/mL in Chik1-ZIF-C, Chik5-ZIF-C and Chik1,5-ZIF-C treated cells^,^ respectively (Figure 4B). In the case of ZIF-C treatment at 6 h. p.i, the reduction in viral titre observed was the lowest in comparison to the other two treatment time points. There is a significant reduction in viral titre from 8.5 log_10_ FFU/mL in VC to 7 log_10_ FFU/mL, 7.2 log_10_ FFU/mL and 6.32 log_10_FFU/mL in Chik1-ZIF-C, Chik5-ZIF-C and Chik1,5-ZIF-C treated cells, respectively. Though significant reductions were observed in the case of CHIKV infected cultures treated with lipofectamine mediated transfection of Chik1-Lipo, Chik5-Lipo, and Chik1,5-Lipo compared to VC, the reduction in virus titre was lower compared to CHIKV infected cell cultures transfected with ZIF-C + siRNA combinations. (Figure 4C). ZIF-C and lipofectamine, when used alone for treatment of infected cells, no reduction in virus titre was observed (Supplementary Figure S4 and Supplementary Figure S5).

**FIGURE 5:**
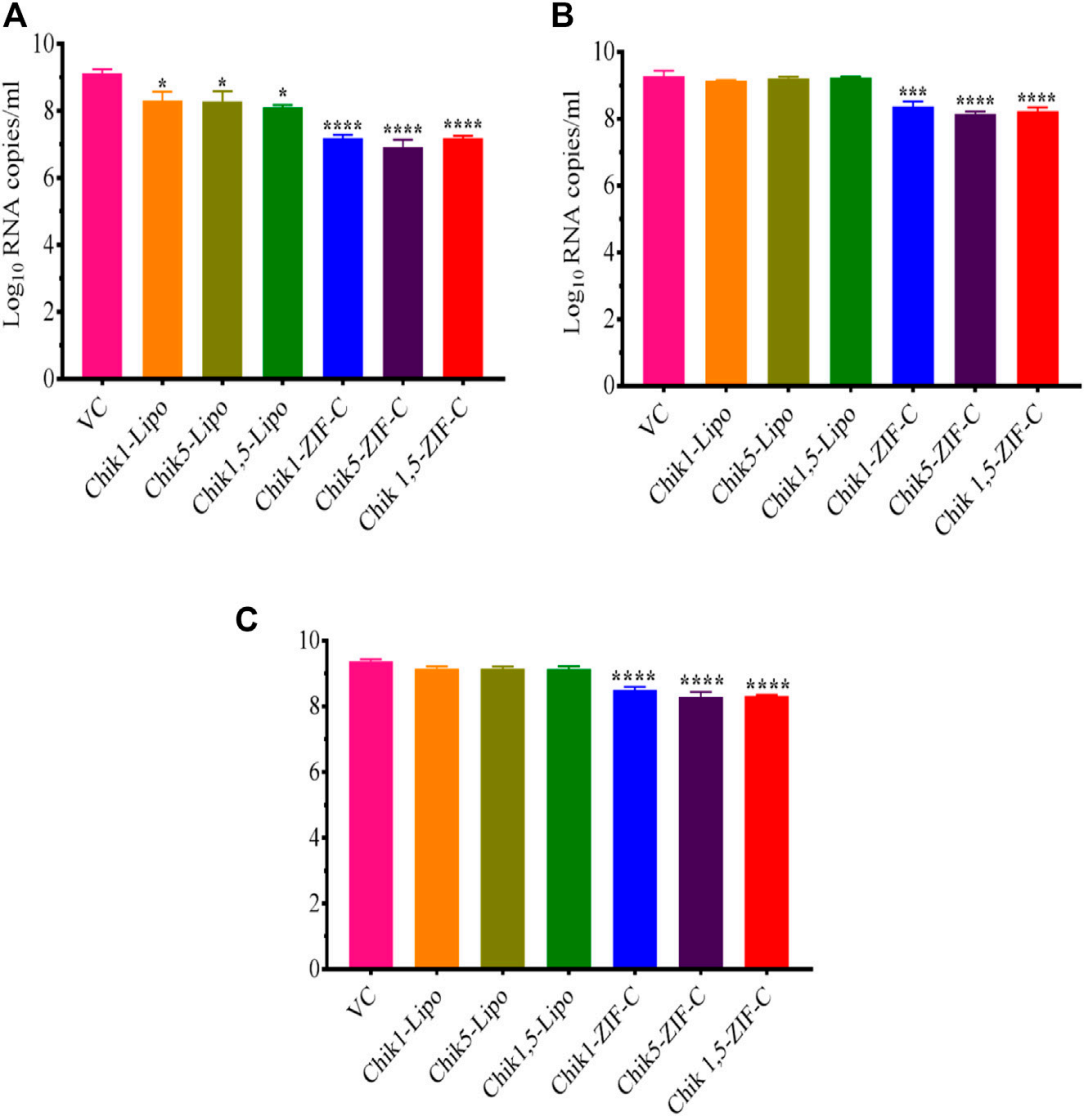
Inhibitory Effect of siRNA-ZIF-C formulations and lipofectamine based siRNA transfection on CHIKV RNA levels quantified by qRT-PCR at treatment after 0 h. p.i, **(A)** 3 h. p.i and **(B)** 6 h. p.i **(C)**. For qRT-PCR, total RNA was extracted and the CHIKV RNA copy number was measured using real-time RT-PCR. The experiments were performed in triplicates in three independent trials. All the values are expressed as mean ± SEM of three experiments. The viral RNA copy number was compared between VC and different siRNA formulations using one-way ANOVA with multiple corrections (****p < 0.0001; ***p < 0.001).

The culture filtrate was investigated by qRT-PCR to decipher the effect of siRNA delivered by different methods into CHIKV infected Vero CCL-81 cells on viral RNA synthesis. In the case of siRNA-ZIF-C treatment at 0 h.p.i, a significant two-log reduction was observed across all the three different ZIF-C-siRNA formulations compared to VC (Figure 5A). The viral RNA copy number reduced from 9.1 log_10_ RNA copies/mL in VC to 7.2 log_10_ RNA copies/mL, 7.3 log_10_ RNA copies/mL and 6.9 log_10_ RNA copies/mL in Chik1-ZIF-C, Chik5-ZIF-C and Chik1,5-ZIF-C formulations, respectively. When ZIF-C-siRNA treatment was carried out 3 h. p.i, a significant one log reduction from 9.3 log_10_ RNA copies/mL in VC to 8.2 log_10_ RNA copies/mL, 8.3 log_10_ RNA copies/mL and 8.1 log_10_ RNA copies/mL for Chik1-ZIF-C, Chik5-ZIF-C and Chik1,5-ZIF-C formulations, respectively was observed (Figure 5B). A similar one-log reduction was noticed in the case of treatment with siRNA-ZIF-C formulations at 6 h. p.i as well (Figure 5C). In the case of lipofectamine-based transfection, a significant reduction of mean log_10_ viral RNA copies/mL was observed from 9.1 log_10_ RNA copies/mL in VC to 8.3 log_10_ RNA copies/mL, 8.4 log_10_ RNA copies/mL and 8.2 log_10_ RNA copies/mL in case of Chik1-Lipo, Chik5-Lipo and Chik1,5-Lipo treatments, respectively (Figure 5A). In the case of lipofectamine mediated siRNA treatment at 3 h.p.i and 6 h.p.i, a mild reduction in log_10_ viral RNA copies/mL was observed compared to VC (Figures 5B, C).

**FIGURE 6:**
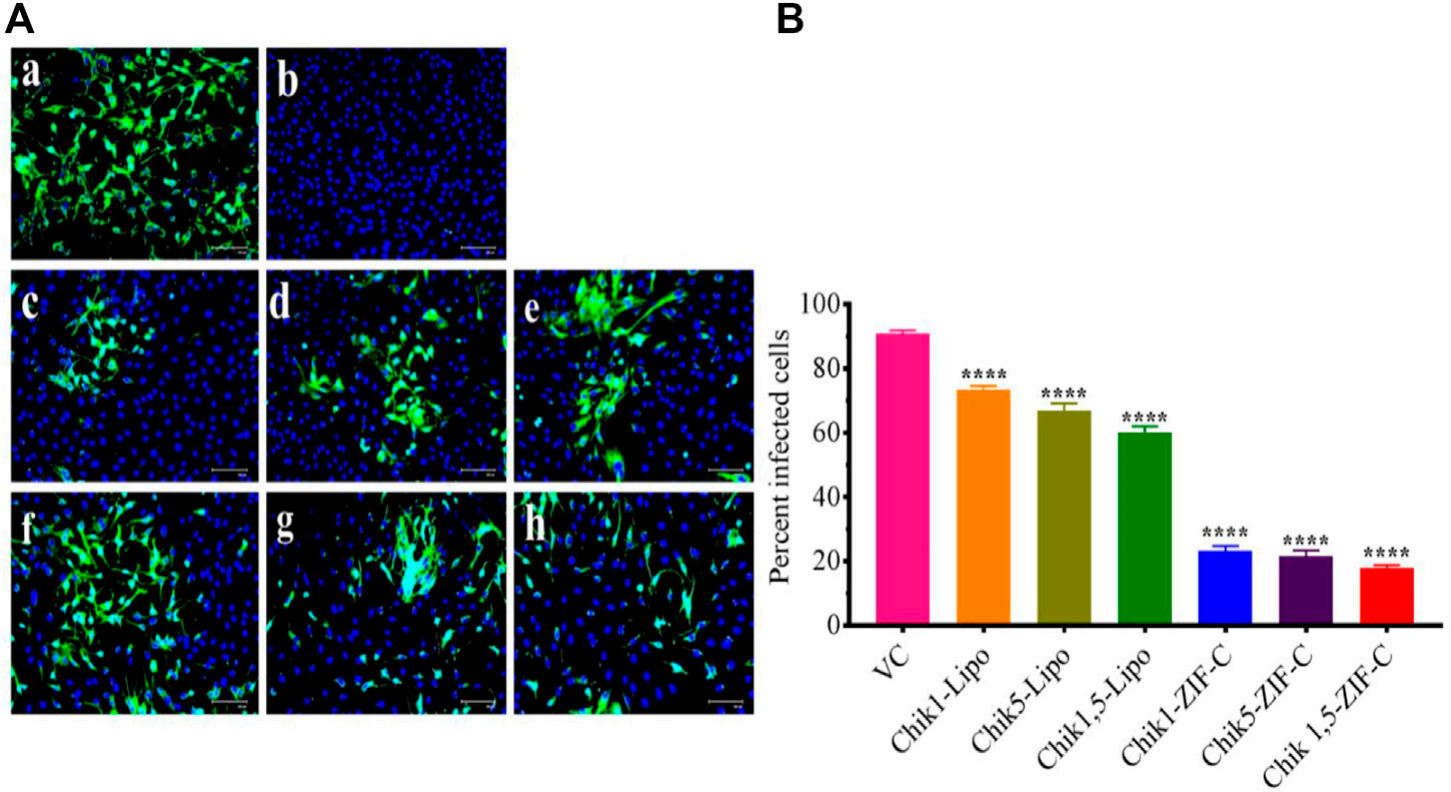
Effect of ZIF-C-siRNA formulations and lipofectamine mediated siRNA transfection on CHIKV infection. Immunofluorescent images **(A)** of Virus Control **(A)**; Cell Control **(B)**; Chik1-ZIF-C **(C)**; Chik5-ZIF-C **(D)**; Chik1,5-ZIF-C **(E)**; Chik1-Lipo **(F)**; Chik5-Lipo **(G)**; Chik1,5-Lipo **(H)** at 0 h.p.i. Virus infected cells are stained green by FITC and the nucleus of the cells is stained blue by DAPI **(A)**. Percent of infected cells during ZIF-C siRNA and Lipofectamine 2000 siRNA treatment (at 0 h. p.i). Cells were counted in four different fields to obtain the percentage of infected cells **(B)**. The experiments were performed in triplicates in three independent trials. All the values are expressed as mean ± SEM of three experiments. The percent infected cells were compared between VC and different siRNA formulations using one-way ANOVA with multiple corrections (****p < 0.0001).

IFA was performed to detect the CHIKV antigen in the infected cells as an indicator of the spread of infection and to determine the extent of inhibition by Chik1 and Chik5 mediated RNAi. IFA was performed only for the treatment at 0 h.p.i time-point. The infected cells were treated with either ZIF-C-siRNA formulation or lipofectamine mediated transfection of siRNA and the cells were incubated for 12 h. After incubation, the cells were processed for staining by fluorescent antibodies and observed under the microscope (Figure 6A). Captured microscopic images were used for counting the infected and uninfected cells using ImageJ software (Rasband 2018). In the case of VC, 93% of cells were observed to be infected while in cultures treated with ZIF-C-siRNA formulations, only 19.6%, 23.9%, and 22.6% of cells were found to be infected in Chik1-ZIF-C, Chik5-ZIF-C and Chik1,5-ZIF-C treatments, respectively (Figure 6B). In the case of lipofectamine mediated delivery of siRNA, a lower reduction in infection percentage was observed i.e., 73.6%, 66.7%, and 60% in Chik1-Lipo, Chik5-Lipo, and Chik1,5-Lipo, respectively compared to VC.

## Discussion

The absence of a licensed vaccine or therapeutic drug for the treatment of CHIKV and its re-emergence in an epidemic form in both tropical, sub-tropical, and temperate regions is a major health concern (Battisti et al., 2021). While the fatality rate is not that high, the effects of chronic CHIKV infection leading to life-long debilitating arthralgia and myalgia along with certain neurological complications observed in certain cases raises the urgency for the development of a potent therapeutic strategy aimed at managing acute and chronic CHIKV infections. Through extensive research using specific chemical compounds, some have been found to have antiviral activity against CHIKV and are being studied further regarding its utility for treatment (Couderc et al., 2009; Delogu et al., 2011; Bourjot et al., 2012; Parashar and Cherian 2014; Kumar et al., 2021). RNA interference especially siRNA-based gene silencing has come up as a potential strategy for the treatment of different kinds of disorders and diseases including viral infections (Levanova and Poranen, 2018). Our previous study documented that siRNA namely Chik1, Chik5, and a combination of both displayed inhibitory effects against CHIKV infection and replication in Vero E6 cell line and C57BL/6 adult mice, respectively (Parashar et al., 2013). The E2 protein is essential for CHIKV entry and assembly inside infected cells while the nsP1 protein is involved in methylation and capping of positive sense viral genome RNA protecting it from degradation and also is crucial for initiation of the negative strand synthesis essential for CHIKV replication (Ahola and Merits, 2016; Metz and Pijlman, 2016). The highly conserved nature of nsP1 and E2 across all CHIKV strains thus bolstered the selection of these two genes as the target genes. The effects medited by siRNA are very specific. Our earlier study has shown that siRNA against dengue and chandipura viruses are not effective in reducing CHIKV titre (Parashar et al., 2013). However, the major problem still hindering the advance of the therapeutic strategy of RNAi is the efficient delivery, uptake, and stability of the desired oligonucleotide in the host system (Castanotto and Rossi, 2009). Physical methods used to transfect target cells or tissue like electroporation, biolistics, laser irradiation, etc. present several issues like poor stability and pharmacokinetic ability of siRNA and other biological effects like off-target effects and interferon response (Meng and Lu, 2017). The lack of an efficient delivery system to target and deliver the siRNA to the desired cells is of particular limitation for the full therapeutic potential of this approach.

Among the different available delivery systems, MOFs show certain unique characteristics such as the facile modification of their inorganic clusters and/or organic ligands to achieve desirable physical (pore size, shape, etc.) and chemical properties (Yan 2017). The moderate strength of its coordination bonds facilitates biodegradation (Sun et al., 2020). A very unique characteristic of MOFs is their ability to interact with biological systems based on various stimuli encountered within the system, which leads to precise and sustained drug release capacity and improved solubility of amorphous and poorly soluble drugs under variable conditions such as pH, light, temperature, pressure, magnetic, glucose level, and multiple stimuli-responsive systems (Jeyaseelan et al., 2021; Mallakpour et al., 2022). The zeolite imidazole framework (ZIF) is a MOF made up of Zn^2+^ and imidazole or its derivatives, and it is the most commonly used drug carrier in Zn-base MOFs. ZIF-8 is the most studied ZIF biocomposite, which is formed by the coordination of Zn^2+^ and the N atom on the 2-methylimidazole ring. Because ZIF-8 is acid-sensitive and has a pH-responsive drug release function, it performs exceptionally well in controlled drug release (Wang et al., 2020).

The present work on ZIF-C mediated delivery of siRNA targeted against CHIKV genes into CHIKV infected Vero CCL-81 cells has shown promising results. Preparation of ZIF-C is a facile and straightforward method. The Chik1 and Chik5 siRNAs and imidazole mixtures turned cloudy immediately after the addition of aqueous Zn^2+^ ions, which indicated the formation of ZIF-based bio-composites. After washing with water, the synthesized biocomposite achieves a ZIF-C crystal phase which has typical aggregated plate-like morphology (Figure 1B) that is in agreement with our published work (Poddar et al., 2020). Our earlier studies suggest that this biomimetic mineralisation approach leads to the encapsulation of >80% nucleic acid within the MOF (Poddar et al., 2020). Therefore, the Chik1 was chosen as a representative siRNA sequence to understand the physicochemical properties of the ZIF-C-based bio-composites. Like other MOFs, ZIF-C also offers maximum protection of loaded nucleic acid from nuclease attack and retains the framework structure in physiological conditions (pH 7.4) (Poddar et al., 2020). However, the Zn-N coordination breaks in an acidic environment (pH < 6) which lead to the release of the loaded nucleic acid (Poddar et al., 2019). After being released, the delivered nucleic acid becomes available in the cell for further actions.

Cell viability assays demonstrated the minimal cytotoxicity of the ZIF-C bio-composites with cell viability greater than 90% as was shown in earlier studies (Poddar et al., 2020). FFU assays to measure infectious viral particles in culture filtrate from the infected cells treated with ZIF-C siRNA formulations demonstrated a significant reduction of viral titer with a ≥99% reduction in the case of all formulations treated CHIKV infected cultures compared to VC. The observation that SARS-CoV-2 siRNA-ZIF-C formulation had no effect on CHIKV titre suggests that the reduction in virus titre is due to the delivered siRNA rather than ZIF-C itself. This reduction of active viral load is attributed to siRNA mediated RNAi of E2 and nsP1 genes of CHIKV resulting in inhibition of replication of CHIKV inside infected cells and hindering the formation and release of progeny virion (Parashar et al., 2013; Ahola and Merits, 2016; Metz and Pijlman, 2016). The effect was maximum when the treatment was given immediately post-infection and with an increasing delay between infection and treatment, the reduction obtained in viral load decreased consistently. This can be attributed to the rapid replicating nature of the CHIKV, which is released into the culture supernatant at a very fast rate leading to further infection of healthy cells in the surrounding environment (Strauss and Strauss, 1994). The results of FFU assays were further corroborated by qRT-PCR results, which revealed a reduction in viral RNA copies per mL in culture filtrates of CHIKV infected cells treated with all three combinations of ZIF-C-siRNA formulations with maximum reduction in cells treated with the combination of both siRNAs. Results of the IFA assay further stand to clarify the results of the RT-PCR and FFU assay. The inhibition of CHIKV replication by Chik1 and Chik5 siRNA, as shown previously by Parashar et al. (2013), is demonstrated by the 65-70 percent reduction in infection percentage of cells when treated with Chik1-ZIF-C, Chik5-ZIF-C, and Chik1,5-ZIF-C in comparison to the VC. The study can be further augmented by assessing the level of reduction in expression of the E2 and nsP1 proteins. This would also provide an idea as to what point in the replication cycle the virus is inhibited. The possible immunogenic effect of ZIF-C also needs to evaluated in *vivo* models since the Vero CCL-81 cell line used in this study is interferon deficient. Lipofectamine mediated transfection of identical combinations of siRNA failed to demonstrate a significant reduction in CHIKV replication compared to VC as well as infected cells treated with ZIF-C siRNA formulations. It might be due to the concentration of Lipofectamine 2000 used to transfect the siRNA might not be enough to serve as an effective carrier as a result of which, adequate levels of siRNA could not be delivered to the cells to initiate RNAi. Due to the restrictions of cytotoxicity (∼15–20%) in Vero CCL-81 cells, the concentration of Lipofectamine 2000 used could not be increased any further (Jeengar et al., 2022).

## Conclusion

To conclude, this study indicates the efficiency of ZIF-C MOF as an efficient delivery system for the delivery of siRNA to inhibit CHIKV replication and further infection in Vero CCL-81 cells. The present study opens new avenues for therapeutic targeting of viral infections using MOF-mediated delivery of siRNAs and further animal studies are needed to confirm *in-vivo* efficacy of the ZIF-C-siRNA.

## Data Availability

The original contributions presented in the study are included in the article/Supplementary Material, further inquiries can be directed to the corresponding authors.

## Supplementary material

Supplementary Figure S1 TEM image of Chik1-ZIF-C.

Supplementary Figure S2 Zn standard curve (ppm vs intensity) to determine concentration of Zn in the prepared Chik-ZIF-C.

Supplementary Figure S3 EDX and elemental map of Chik1-ZIF-C.

Supplementary Figure S4 Inhibitory effects of siRNA-ZIF-C and only ZIF-C on production of virus particles as assessed by FFU assay (**** *p* < 0.0001).
