# Supplementary figures and images for: Targeted in vitro gene silencing of E2 and nsP1 genes of chikungunya virus by biocompatible zeolitic imidazolate framework
Source: Front Bioeng Biotechnol. 2022 Dec 14;10:1003448. doi: 10.3389/fbioe.2022.1003448 (PMC9806579; doi:10.3389/fbioe.2022.1003448)

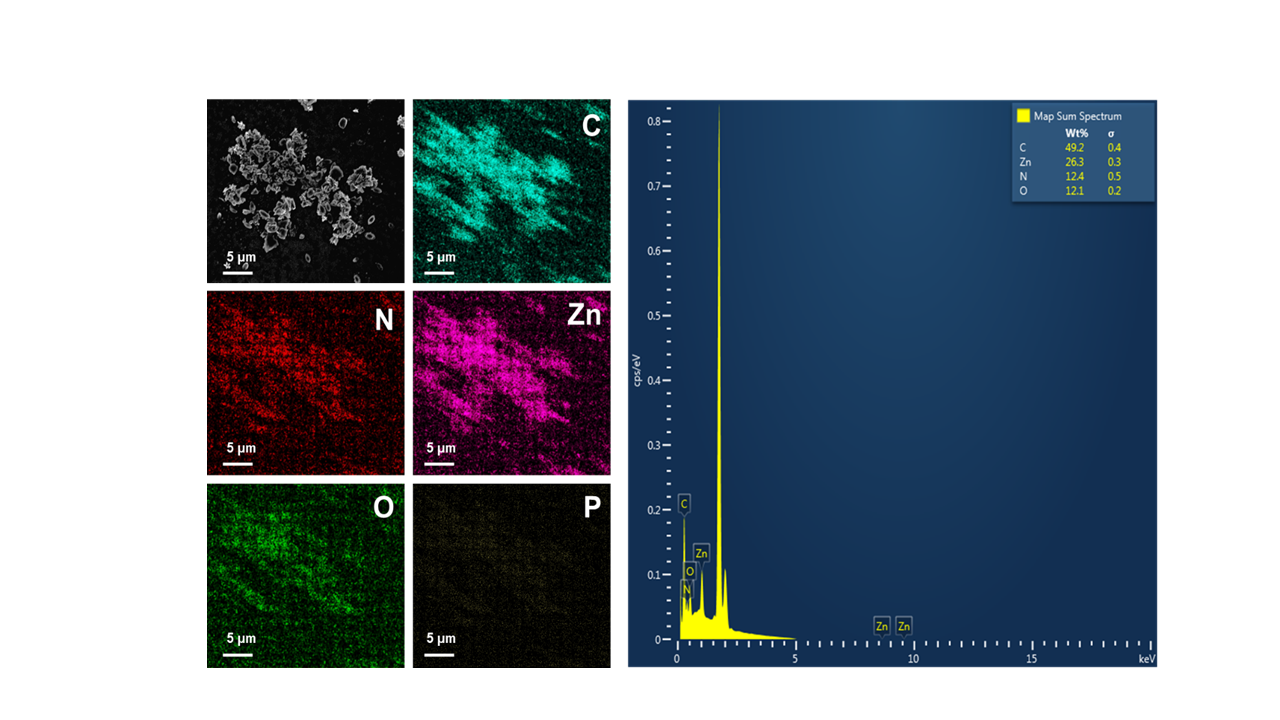

Supplement: Supplementary file 1 [file Image3.TIFF]

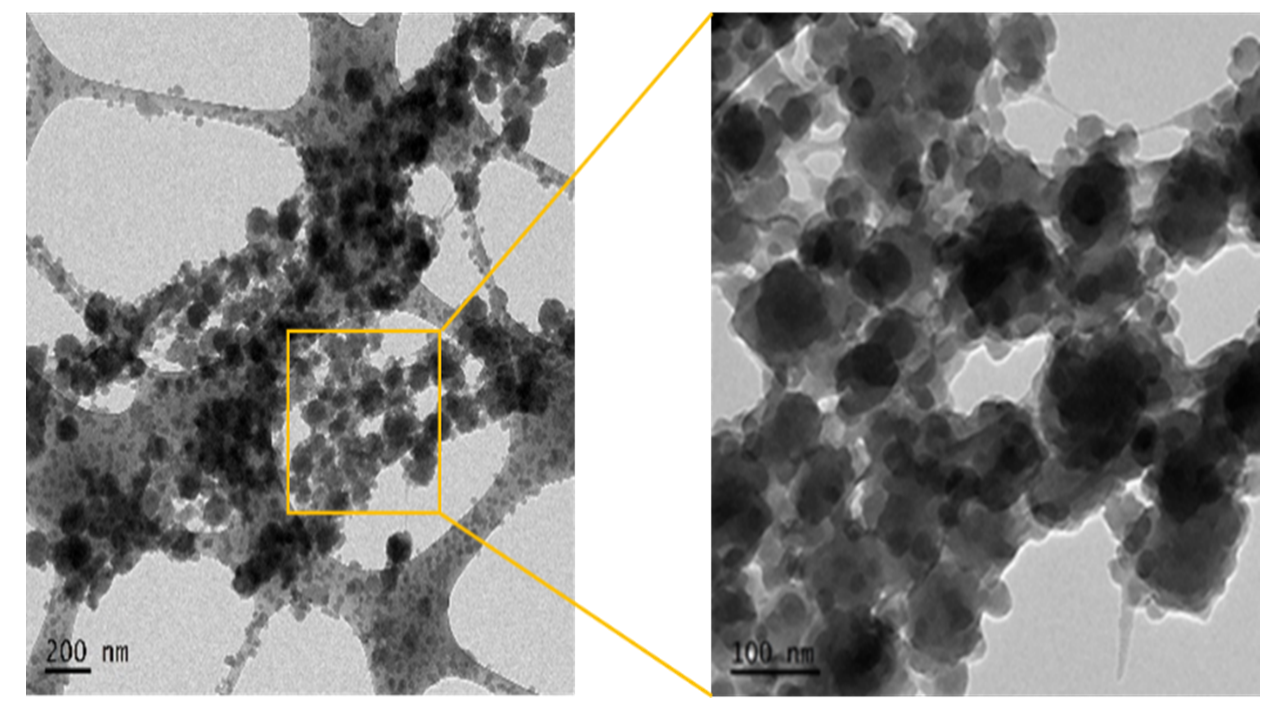

Supplement: Supplementary file 2 [file Image1.TIFF]

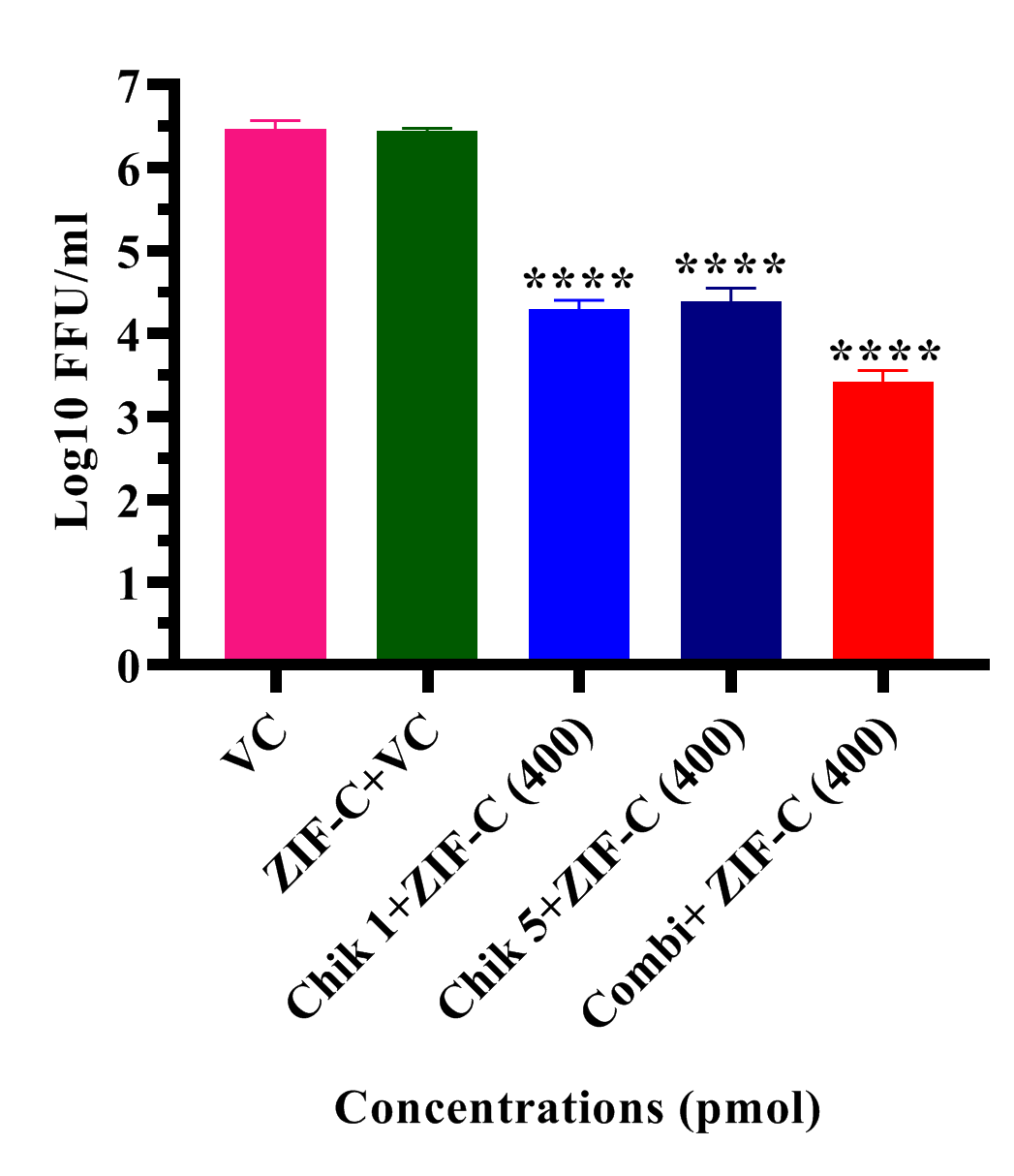

Supplement: Supplementary file 3 [file Image4.TIF]

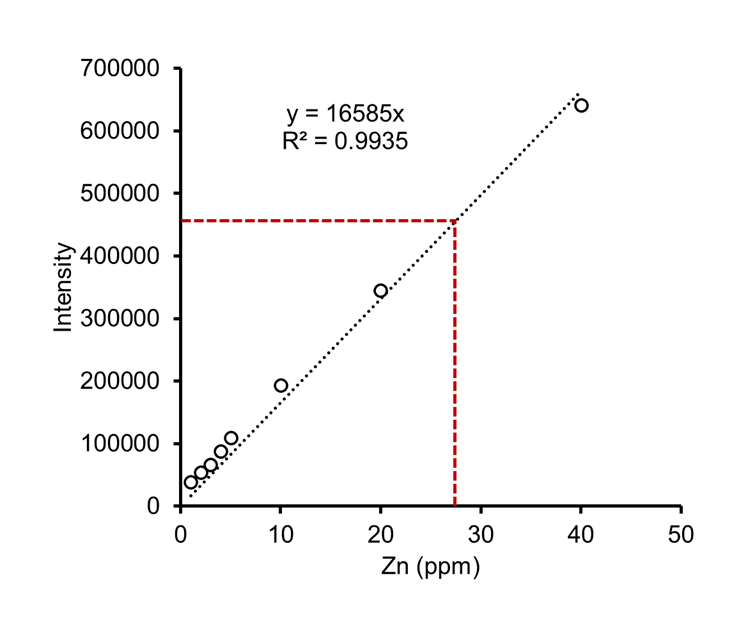

Supplement: Supplementary file 4 [file Image2.TIF]

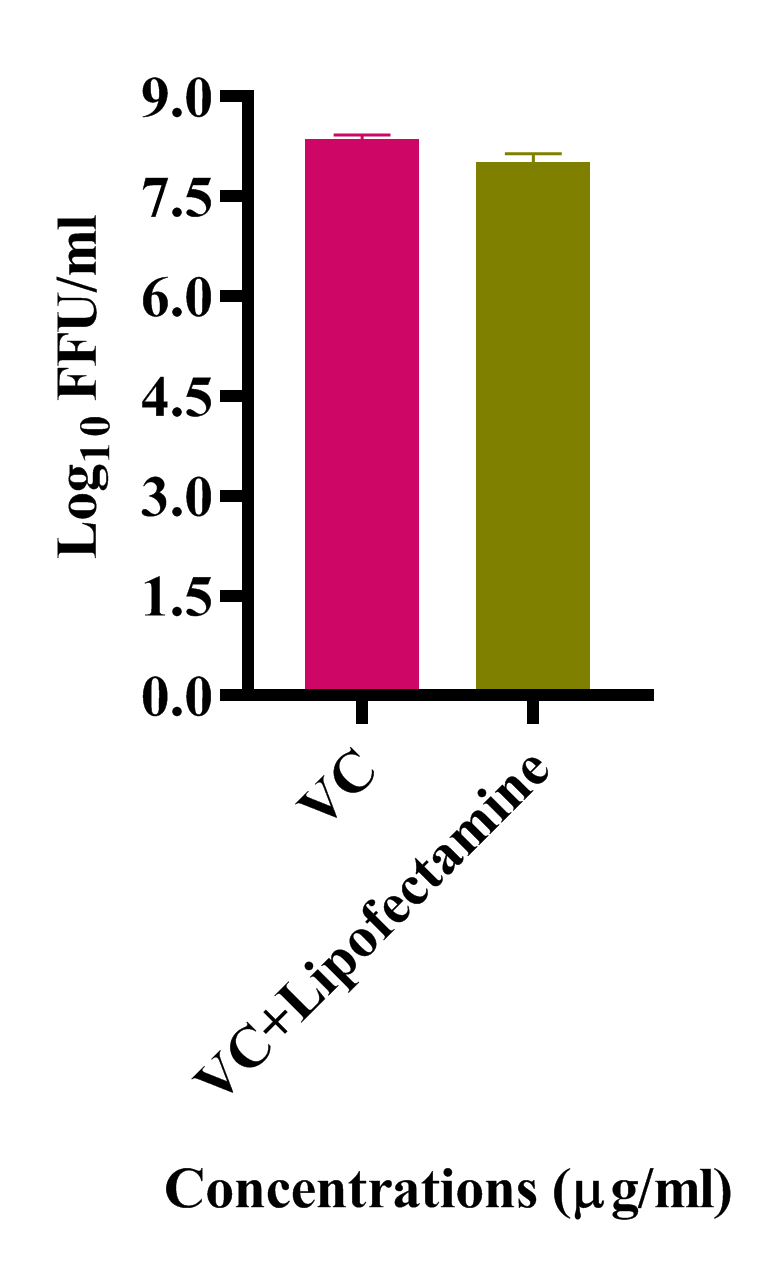

Supplement: Supplementary file 5 [file Image5.TIF]
